# Protocol for the postoperative radiotherapy in N1 breast cancer patients (PORT-N1) trial, a prospective multicenter, randomized, controlled, non-inferiority trial of patients receiving breast-conserving surgery or mastectomy

**DOI:** 10.1186/s12885-022-10285-0

**Published:** 2022-11-16

**Authors:** Tae Hoon Lee, Ji Hyun Chang, Bum-Sup Jang, Jae Sik Kim, Tae Hyun Kim, Won Park, Yong Bae Kim, Su Ssan Kim, Wonshik Han, Han-Byoel Lee, Kyung Hwan Shin

**Affiliations:** 1 Department of Radiation Oncology, Seoul National University Hospital, 101, Daehak-ro, Jongno-gu, Seoul, 03080 Republic of Korea; 2 Department of Radiation Oncology, Seoul National University College of Medicine, Seoul, Republic of Korea; 3 Department of Radiation Oncology, Soonchunhyang University Seoul Hospital, Seoul, Republic of Korea; 4 Department of Radiation Oncology, Research Institute and Hospital, National Cancer Center, Goyang, Republic of Korea; 5 Department of Radiation Oncology, Samsung Medical Center, Sungkyunkwan University School of Medicine, Seoul, Republic of Korea; 6 Department of Radiation Oncology, Yonsei Cancer Center, Yonsei University College of Medicine, Seoul, Republic of Korea; 7 Department of Radiation Oncology, Asan Medical Center, University of Ulsan College of Medicine, Seoul, Republic of Korea; 8 Department of Surgery, Seoul National University Hospital, Seoul National University College of Medicine, Seoul, Republic of Korea; 9 Cancer Research Institute, Seoul National University College of Medicine, Seoul, Republic of Korea

**Keywords:** Adjuvant radiotherapy, Breast cancer, Lumpectomy, Mastectomy, pN1

## Abstract

**Background:**

Postoperative radiotherapy (PORT) could be useful for pN1 breast cancer patients who have undergone breast-conserving surgery (BCS) or mastectomy. However, the value of regional nodal irradiation (RNI) for BCS patients, and the indications for post-mastectomy radiotherapy (PMRT) for pN1 breast cancer mastectomy patients, have recently been challenged due to the absence of relevant trials in the era of modern systemic therapy. “PORT de-escalation” should be assessed in patients with pN1 breast cancer.

**Methods:**

The PORT-N1 trial is a multicenter, randomized, phase 3 clinical trial for patients with pN1 breast cancer that compares the outcomes of control [whole-breast irradiation (WBI) and RNI/PMRT] and experimental (WBI alone/no PMRT) groups. PORT-N1 aims to demonstrate non-inferiority of the experimental group by comparing 7-year disease-free survival rates with the control group. Female breast cancer patients with pT1-3 N1 status after BCS or mastectomy are eligible. Participants will be randomly assigned to the two groups in a 1:1 ratio. Randomization will be stratified by surgery type (BCS vs. mastectomy) and histologic subtype (triple-negative vs. non-triple-negative). In patients who receive mastectomy, dissection of ≥5 nodes is required when there is one positive node, and axillary lymph node dissection when there are two or three positive nodes. Patients receiving neoadjuvant chemotherapy are not eligible. RNI includes a “high-tangent” or wider irradiation field. This study will aim to recruit 1106 patients.

**Discussion:**

The PORT-N1 trial aims to verify that PORT de-escalation after BCS or mastectomy is safe for pN1 breast cancer patients in terms of oncologic outcomes and capable of reducing toxicity rates. This trial will provide information crucial for designing PORT de-escalation strategies for patients with pN1 breast cancer.

**Trial registration:**

This trial was registered at ClinicalTrials.gov (NCT05440149) on June 30, 2022.

## Background

Although surgery is the mainstay of definitive breast cancer treatment, postoperative radiotherapy (PORT) also plays an essential role. The two main types of radical surgery for breast cancer are breast-conserving surgery (BCS) and mastectomy. PORT is crucial in most patients who have undergone BCS: several randomized trials and meta-analyses showed that PORT reduces breast cancer recurrence and death [1, 2]. However, PORT is not always the best option for patients who received a mastectomy. Radiotherapy is recommended when the tumor is large (> 5 cm) or regional metastasis is confirmed during axillary surgery [3, 4]. These principles have informed breast cancer treatment for many years.

Systemic therapy is another essential component of breast cancer treatment, and has advanced greatly in recent years. Long-term hormonal therapy and ovarian suppression have been applied in patients with positive hormonal receptors to further decrease the risk of breast cancer recurrence [5, 6]. Trastuzumab is widely used in human epidermal growth factor receptor 2 (HER2)-positive breast cancer patients and significantly improves the prognosis [7]. High-risk HER2-positive breast cancer patients can also benefit from pertuzumab [8]. The addition of taxane to adjuvant chemotherapy has increased patient survival rates [9]. However, as the major clinical trials supporting PORT did not consider these advancements [10], it remains uncertain whether modern systemic therapy impacts the effectiveness of radiotherapy. With the increased application of systemic therapy, de-escalation of certain aspects of PORT may be feasible in selected patients, such as field size reduction or the omission of radiotherapy.

The PORT field is subject to debate. A larger radiation field is needed for patients at higher risk of regional metastasis; however, the extent of the field and factor necessitating a larger one have not been determined, especially in patients with 1–3 positive axillary lymph nodes. Some randomized trials have attempted to determine the effectiveness of regional nodal irradiation (RNI). In the MA.20 trial, which included breast cancer patients with high-risk pathologic features, RNI covered axillary, supraclavicular lymph node (SCL), and internal mammary lymph node (IMN) areas. The results showed benefits of RNI in terms of disease-free survival (DFS) [11]. In the European Organisation for Research and Treatment of Cancer (EORTC) 22922/10925 trial, irradiation of the IMN and medial SCL areas was performed, and breast cancer mortality was decreased [12]. The IMN was also irradiated in a French trial, but there was no survival benefit [13]. As extent of RNI was different between these randomized trials, indications for irradiation of specific nodes cannot be established [14]. Furthermore, the indications of RNI according to the number of positive lymph nodes in axillary surgery remain uncertain.

The current indications for PORT after mastectomy have been criticized. Although the Early Breast Cancer Trialists’ Collaborative Group meta-analysis confirmed a benefit of post-mastectomy radiotherapy (PMRT) for pN1 patients [15], this conclusion was based primarily on the results of two Danish trials criticized for their high rates of inadequate axillary surgery. In addition, more than two decades have passed since these results were reported, and the benefit of PORT may have lessened due to advances in breast cancer treatment [10]. Secondary analysis of the prospective data of pT1-2 N1 patients enrolled in the BIG 02-98 randomized trial of adjuvant chemotherapy revealed that PMRT slightly improved locoregional control, but not survival rates [16]. Thus, the role of PORT after mastectomy must be reevaluated based on more recent evidence.

Although several non-randomized series have provided insight into “PORT de-escalation” [17–19], oncologic safety has not been confirmed. If the oncologic safety of PORT de-escalation can be established, breast cancer patients may benefit from reduced toxicity and lower time and medical costs. Accordingly, the Korean Radiation Oncology Group (KROG) designed a multicenter clinical trial of PORT for patients with pN1 breast cancer: the Postoperative Radiotherapy in N1 Breast Cancer Patients (PORT-N1) trial. This trial aims to evaluate the feasibility of PORT de-escalation in pN1 breast cancer patients by establishing indications for RNI in patients receiving BCS, and for PMRT in patients receiving a mastectomy.

## Methods and design

### Study design

**Fig. 1:**
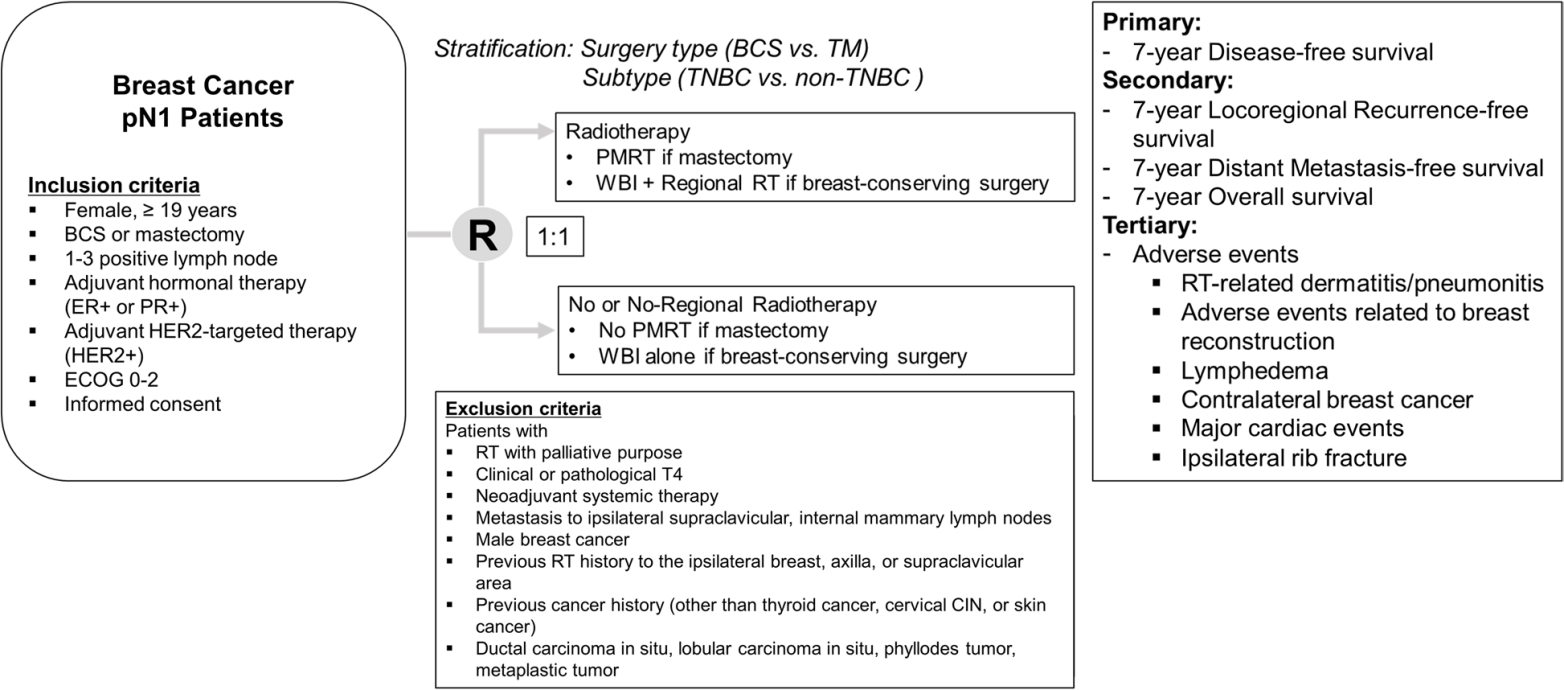
Schematic diagram of the design of the Postoperative Radiotherapy in N1 Breast Cancer Patients (PORT-N1) trial of patients receiving breast-conserving surgery (BCS) or mastectomy. CIN, cervical intraepithelial neoplasia; ECOG, Eastern Cooperative Oncology Group; ER, estrogen receptor; HER2, human epidermal growth factor receptor 2; PMRT, post-mastectomy radiotherapy; PR, progesterone receptor; TM, total mastectomy; RT, radiation therapy; TNBC, triple-negative breast cancer; WBI, whole-breast irradiation

PORT-N1 is a prospective multicenter randomized controlled non-inferiority trial. The study design is illustrated in Fig. 1. Twenty-one academic centers in South Korea are participating in this trial. Participants with 1–3 positive lymph nodes (found during axillary surgery), who did not receive neoadjuvant systemic therapy and underwent BCS or mastectomy, were randomized in a 1:1 ratio to the control and experimental groups. Participants allocated to the control group who underwent BCS will undergo whole-breast irradiation (WBI) with RNI, while those who underwent mastectomy will undergo PMRT. Participants allocated to the experimental group will undergo WBI only if they underwent BCS. PMRT will not be performed in experimental group participants who underwent mastectomy. This trial is anticipated to run for 11 years, with a 4-year recruitment phase and 7-year follow-up. The first participant was recruited on July 19, 2022. Participant registration is expected to be completed in December 2026, and the final results will be available after 2033. This protocol was approved by the KROG and designated as KROG 22-05. This trial was registered at ClinicalTrials.gov (NCT05440149) on June 30, 2022.

### Eligibility criteria

The inclusion criteria are as follows: female patients aged ≥19 years living in South Korea available for long-term follow-up; receiving BCS or mastectomy for pathologically confirmed invasive breast cancer; 1–3 positive axillary lymph nodes (of any diameter) confirmed during the pathologic examination of a surgical specimen, with the additional requirements of ≥5 lymph nodes dissected in cases with 1 positive lymph node and axillary lymph node dissection in cases with 2–3 positive lymph nodes for mastectomy; adjuvant hormonal therapy scheduled for a positive estrogen or progesterone receptor; adjuvant HER2-targeted therapy scheduled for HER2-positive cancer; Eastern Cooperative Oncology Group (ECOG) performance status of 0–2; and provision of written informed consent.

The exclusion criteria are as follows: radiotherapy scheduled for palliative purposes; T4 disease revealed by clinical or pathologic examination; receiving neoadjuvant systemic treatment; regional metastasis in the ipsilateral SCL or IMN area, or distant metastasis; male breast cancer patient; prior history of ipsilateral breast, axilla, or SCL irradiation; prior history of other cancers (excluding thyroid cancer, carcinoma in situ of uterine cervix, and skin cancer); and ductal or lobular carcinoma in situ, phyllodes tumor, or metaplastic tumor diagnosed during pathologic examination of a breast surgical specimen.

### Sample size calculation

The PORT-N1 trial was designed to determine whether the omission of RNI from BCS and PMRT in patients undergoing a mastectomy reduced treatment efficacy. The expected 7-year DFS of the control group in this trial is 90%, based on a previous multicenter retrospective studies conducted by the KROG. The rate of recurrence at 7 years was 12% for the no-PMRT group in KROG 14-23 [20]; the recurrence rate is expected to be lower in the PORT-N1 trial because more than a decade has passed since the previous study. The KROG 14-18 study analyzed pN1 breast cancer patients who underwent anthracycline and taxane chemotherapy, and reported a 5-year DFS of 92.4%, with no difference seen between BCS plus PORT and mastectomy alone groups [21]. Another analysis of KROG 14-18 data compared WBI alone and WBI plus SCL irradiation, and obtained 5-year DFS rates of 94.4 and 92.6%, respectively [19]. The sample size calculation for the PORT-N1 trial assumes a non-inferiority margin of 5%, type I error rate of 5%, 4-year recruitment period, 7-year follow-up, and exponential distribution for time to progression; 134 DFS events are expected, and 525 patients are required for each group for a power of 80%. Assuming a dropout rate of 5%, the final number of patients to be randomized is 1106 (553 for each group).

### Screening and randomization

Participants will be screened at a radiation oncology department after surgery. After obtaining written informed consent, inclusion and exclusion criteria will be applied based on clinical records, pathologic reports for surgical specimens, and additional history data. Participants who meet the criteria will be enrolled and randomized to the control and experimental groups in a 1:1 ratio. Randomization will be performed by an authorized investigator using a web-based system. The randomization system was devised by the Medical Research Collaborating Center (MRCC) of Seoul National University Hospital and is operated separately from other clinical investigators. Amendment of the randomization process is strictly forbidden by the MRCC. The stratified block randomization method will be used to prepare a randomization table. The stratification factors include surgery type (BCS vs. mastectomy) and histologic subtype (triple-negative vs. non–triple-negative breast cancer). The investigators will submit the screening number, participating institution, date of informed consent, and birthday of the participant to the randomization system to prevent duplicate enrollment. The participants and investigators are not blinded to the randomization results, and the participant may be informed of the precise details and field settings of the radiotherapy. After group allocation is confirmed, the written consent form will be printed and stored with the clinical trial documents.

### Interventions and follow-up

There are no precise requirements for surgical and adjuvant systemic therapy; participants will be treated based on institutional preference, except in the case of radiotherapy. Genomic testing to determine suitability for adjuvant chemotherapy is optional. For PORT, a photon beam of 4–10 MV is recommended. Both three-dimensional conformal radiation therapy and intensity-modulated radiation therapy are permitted for radiotherapy planning and beam delivery. An electron beam can be applied for IMN and tumor bed boost irradiation. Computed tomography-based simulation is required for all participants. Both hypofractionated and standard dose-fractionation schemes are allowed. Hypofractionated regimens are defined as those with a dose per fraction of 2.5–3.0 Gy, dose to the whole breast or chest wall of 39–45.9 Gy, and 13–17 fractions. Standard fractionation regimens are those with a dose per fraction of 1.8–2.0 Gy, dose to whole breast or chest wall of 45–50.4 Gy, and 23–28 fractions. There are no restrictions in terms of the tumor bed boost irradiation technique or dose fractionation scheme.

**Fig. 2:**
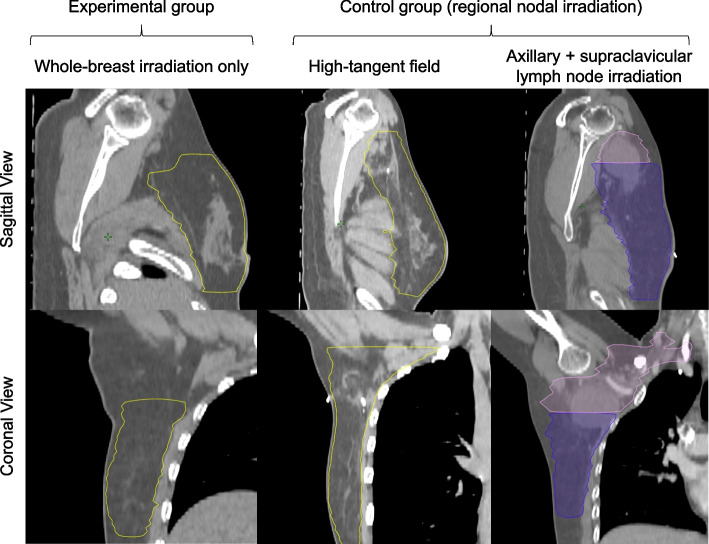
Example clinical target volumes for whole-breast irradiation (WBI) only, high-tangent field irradiation, and axillary and supraclavicular lymph node irradiation. High-tangent field or larger was permitted for postoperative radiotherapy after breast-conserving surgery in the control group (WBI and regional nodal irradiation)

**Fig. 3:**
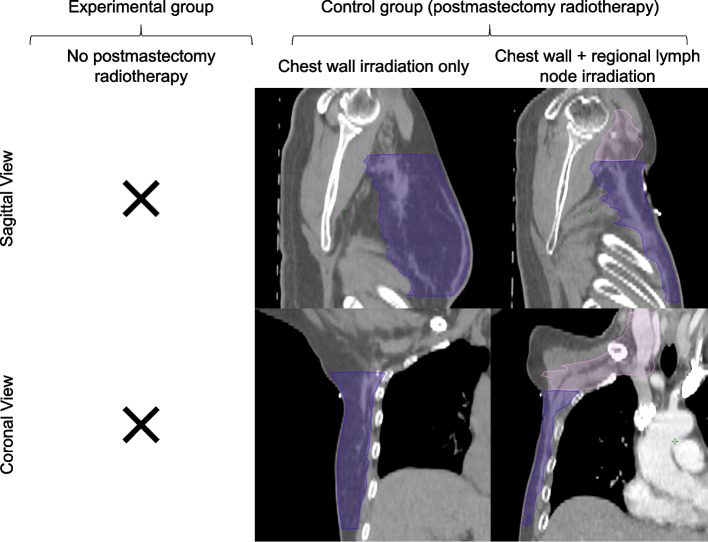
Example clinical target volumes for post-mastectomy radiotherapy. No post-mastectomy radiotherapy is permitted in the experimental group. Chest wall irradiation with or without regional lymph node irradiation is allowed in the control group

The clinical target volume (CTV) should be delineated based on Radiation Therapy Oncology Group (RTOG) [22] or EORTC guidelines [23, 24]. In control group participants who underwent BCS, the whole breast and regional lymph node area should be irradiated. The regional radiation field should be a “high-tangent” (upper margin ≤2 cm inferior to the humeral head, to encompass axillary levels I and II) or wider field. The SCL and IMN areas can be irradiated in the control group. Examples of CTV delineation for PORT after BCS in both groups are shown in Fig. 2. In participants who underwent mastectomy, the chest wall should be irradiated with or without inclusion of the regional lymph node area. In the experimental group, WBI alone (without RNI) will be performed in participants who underwent BCS, and the CTV should be delineated accordingly. No irradiation will be delivered to patients in the experimental group who underwent mastectomy. Examples CTVs for PMRT are shown in Fig. 3. Implant-saving target delineation is allowed, based on published guidelines [25].

Regular follow-up visits will be scheduled (at 6 months and 12 months, and once per year thereafter) for 7 years after the day of randomization. Performance status, disease status, survival, and adverse events will be recorded at every follow-up visit.

### Safety monitoring

Adverse events to be recorded include radiation dermatitis, radiation pneumonitis, major reconstruction surgery complications, lymphedema, contralateral ductal carcinoma in situ or invasive breast cancer, major coronary events, ipsilateral rib fracture, and others. Radiation dermatitis and pneumonitis will be evaluated based on the Common Terminology Criteria for Adverse Events (CTCAE) version 5. Major reconstruction surgery complications are defined as unplanned rehospitalization, reoperation for intervention, implant removal due to infection, and autologous flap failure. Lymphedema will be recorded when the participant complains of lymphedema symptoms or additional symptomatic treatment is required. An adverse event will be recorded when ductal carcinoma in situ or invasive cancer in the contralateral breast is histologically diagnosed. Major coronary events include myocardial infarction, ischemic heart failure, unstable angina, and sudden death. Ipsilateral rib fracture revealed by a radiographic or nuclear medical scan will be reported regardless of symptoms. Other adverse events may be recorded and graded according to CTCAE version 5. Severe adverse events should be reported to the institutional review board within 7 working days. An institutional investigator should report all severe adverse events to the primary investigator. A severe adverse events report will be sent to the institutional review board of each participating center.

### Data management

The principal investigator has full responsibility for the management of clinical trial data. Investigators will complete case report forms using a web-based system (iCReaT version 2) provided by the Korean National Institute of Health. Data monitoring will be performed by a clinical research organization (Promedis Inc., Seoul, Republic of Korea) and the clinical research coordinator of each participating center. Documents generated from this trial will be stored at each participating center until 10 years after trial completion, with approval from the institutional review board, after which personal information will be discarded.

### Endpoint and statistics

The primary endpoint of this trial is 7-year DFS. The secondary endpoints are locoregional recurrence-free survival (LRFS), distant metastasis-free survival (DMFS), and overall survival (OS). A DFS event is defined as breast cancer recurrence or death. An LRFS event is defined as recurrence in a locoregional area, including the ipsilateral breast/chest wall, axilla, IMN, and SCL area, or breast cancer death. A DMFS event is defined as distant metastasis or breast cancer death. For OS, an event is defined as patient death from any cause. These endpoints will be measured from the date of randomization.

Categorical variables will be compared between the control and experimental groups using the chi-square test, and continuous variables using Student’s *t*-test. DFS, LRFS, DMFS, and OS rates will be calculated by Kaplan–Meier analysis and compared using the log-rank test. The Cox proportional hazards model will be used to evaluate the impact of covariates on clinical endpoints. The threshold for statistical significance is *P* < 0.05. No interim analysis is planned for this trial.

## Discussion

The PORT-N1 trial aims to evaluate the indications for RNI in pN1 patients who have undergone BCS, and for PMRT in pN1 patients who have undergone mastectomy. These radiotherapeutic procedures are recommended by institutional guidelines, including those of the National Comprehensive Cancer Network; however, several studies have challenged this. In a prospective series conducted at the Memorial Sloan–Kettering Cancer Center (MSKCC), a low risk of regional recurrence was reported without RNI in patients with cT1-2 N0 breast cancer and one or two positive sentinel lymph nodes [18]. Furthermore, the findings of the secondary analysis of the BIG 02-98 trial [16] and the large MSKCC retrospective series [17] challenge the routine use of PMRT for pN1 breast cancer in patients who have undergone mastectomy. We expect the large prospective PORT-N1 trial to provide robust evidence guiding PORT use in patients with pN1 breast cancer.

Surgical advances prompted attempts to reduce the use and indications for axillary surgery. Sentinel lymph node biopsy alone is recommended over axillary lymph node dissection in many early breast cancer cases [26]. Even with a false-negative rate of approximately 10% for sentinel lymph node biopsy, routine axillary dissection did not improve the outcomes of patients with breast cancer [27]. Axillary dissection after positive sentinel lymph node biopsy can now be omitted for selected patients according to the results of the Z0011 trial [28], and a randomized controlled trial assessing whether sentinel lymph node biopsy can be omitted in patients subjected to preoperative evaluation of the axilla and postoperative axillary irradiation is ongoing (NAUTILUS trial, NCT04303715) [29]. Optimizing the radiation field and indications for PORT would aid the establishment of a robust axillary management strategy for patients.

This protocol establishes a minimum number of dissected lymph nodes as an additional requirement for patients receiving mastectomy, due to concerns about the risk of locoregional recurrence after insufficient axillary dissection with complete PORT omission. Conventionally, the total number of surgically removed lymph nodes is thought to be associated with breast cancer prognosis [30]. However, this concept has been challenged by studies on the de-escalation of axillary dissection [27, 28]. Nevertheless, researchers remain cautious with respect to omitting both axillary dissection and PORT in cases of node-positive breast cancer. Notably, in ongoing trials examining the de-escalation of axillary surgery, further axillary dissection is being replaced with radiotherapy [29, 31].

We expect a lower prevalence of lymphedema to emerge as a benefit of decreasing the radiation field size and indications for PORT. Lymphedema is a major form of toxicity associated with breast cancer treatment. A recent large Korean retrospective study reported a 3-year lymphedema rate of approximately 10% [32], although the incidence may vary among diagnostic methods [33]. Lymphedema significantly affects the quality of life of breast cancer survivors, both physically and psychosocially [34]. Many studies have linked lymphedema risk with surgical factors such as the number of removed lymph nodes [35]. However, several reports have indicated that wider RNI is also associated with a higher incidence of lymphedema [32, 36, 37]. As an additional objective, this trial will assess whether PORT de-escalation impacts the incidence of lymphedema in patients with pN1 breast cancer. Toxicity and oncologic safety data from this trial are expected to greatly influence clinical decision-making.

Another ongoing multicenter, randomized, controlled trial aiming to determine the role of PORT in pN1 breast cancer was initiated by the KROG (KROG 17-01; NCT03269981) [38]. The KROG 17-01 trial has a similar design to the present trial, although there are some critical differences. First, KROG 17-01 includes only BCS patients, whereas this trial includes both BCS and mastectomy patients. Second, an eligibility criterion for KROG 17-01 is the use of taxane chemotherapy as adjuvant treatment, whereas in this trial there is no mandatory chemotherapeutic regimen. Third, in KROG 17-01 RNI mainly covers the axillary, SCL, and IMN areas, although IMN can be excluded according to the preference of individual institutions. In the PORT-N1 trial, RNI has a broader definition, including irradiation to axilla levels I and II only in cases of high tangent field, based on the opinions of several experts who favor a smaller regional radiation field for low-risk pN1 breast cancer patients. Participant recruitment to the KROG 17-01 trial has now closed. We believe that the KROG prospective trials will provide crucial information for the performance of PORT in pN1 breast cancer patients.

Other ongoing trials testing PORT de-escalation include the National Surgical Adjuvant Breast and Bowel Project (NSABP) B-51/RTOG 1304 (NCT01872975) trial, which is a prospective randomized, controlled trial designed to determine whether RNI after BCS and chest wall irradiation with RNI after mastectomy affect the recurrence-free interval [39]. The NSABP B-51/RTOG 1304 and PORT-N1 trials define their experimental and control groups similarly, but differ greatly in terms of their inclusion criteria. Participants of the NSABP B-51/RTOG 1304 trial are those undergoing neoadjuvant chemotherapy and achieving ypN0 status; patients receiving neoadjuvant chemotherapy are excluded in the PORT-N1 trial. The NEONOD2 trial (NCT04019678) is a prospective uncontrolled trial that compares outcomes between ypN1mi and ypN0 breast cancer patients [40]. Neither axillary dissection nor RNI is permitted in either group. The NEONOD2 trial aims to determine whether the omission of axillary dissection and RNI for nodal micrometastasis is feasible after systemic chemotherapy. Along with these trials, PORT-N1 is expected to establish PORT de-escalation strategies for breast cancer.

In conclusion, the PORT-N1 trial aims to verify that PORT de-escalation after BCS or mastectomy in pN1 breast cancer is suitable and lowers toxicity. The non-inferiority results of the current trial suggest a benefit for patients, which supports a reduction of the radiation field after BCS or omission of radiotherapy after mastectomy. Thus, we anticipate that the data collected in this trial will inform a decision process for managing patients with pN1 breast cancer.

## Data Availability

Not applicable.

## Abbreviations

BCS: Breast-conserving surgery
CTCAE: Common Terminology Criteria for Adverse Events
CTV: Clinical target volume
DFS: Disease-free survival
DMFS: Distant metastasis-free survival
ECOG: Eastern Cooperative Oncology Group
EORTC: European Organisation for Research and Treatment of Cancer
HER2: Human epidermal growth factor receptor 2
IMN: Internal mammary lymph node
KROG: Korean Radiation Oncology Group
LRFS: Locoregional recurrence-free survival
MRCC: Medical Research Collaborating Center
MSKCC: Memorial Sloan–Kettering Cancer Center
NSABP: National Surgical Adjuvant Breast and Bowel Project
OS: Overall survival
PMRT: Post-mastectomy radiotherapy
PORT: Postoperative radiotherapy
RNI: Regional nodal irradiation
RTOG: Radiation Therapy Oncology Group
SCL: Supraclavicular lymph node
WBI: Whole-breast irradiation
